# Rate of advance care planning practice during the COVID-19 outbreak in Japan: a cross-sectional survey study

**DOI:** 10.1007/s40520-025-03004-9

**Published:** 2025-04-07

**Authors:** Hungu Jung, Masahiro Akishita, Yuji Iwamoto, Junpei Tanabe, Kenta Hirohama, Shinya Ishii

**Affiliations:** 1 Department of Medicine for Integrated Approach to Social Inclusion, Graduate School of Biomedical and Health Sciences, Hiroshima University, 1-2-3 Kasumi, Minami-Ku, Hiroshima City, Hiroshima Japan; 2 Tokyo Metropolitan Institute for Geriatrics and Gerontology, 35-2 Sakae-cho, Itabashi-ku, Tokyo, Japan

**Keywords:** Advance care planning, COVID-19, Dementia, Geriatricians, Patients

## Abstract

**Background:**

Advance care planning (ACP) ensures that future care is provided during serious illness, considering an individual’s wishes; it is particularly important for older adults. Regarding ACP practices during the coronavirus disease (COVID-19) outbreak, although there are reports on patients, reports on geriatricians are scarce.

**Aim:**

This study evaluated the rate of ACP practice during the COVID-19 outbreak through a questionnaire survey of geriatricians.

**Methods:**

This cross-sectional study surveyed geriatric specialists, who were members of the Japanese Geriatric Society, between October and December 2022 using an anonymous online questionnaire. The questionnaire covered the treatment of COVID-19 patients, difficulties encountered when caring for older patients with COVID-19 infection, and COVID-19 sequelae. Multiple logistic regression with a forward stepwise method was performed to determine the factors associated with ACP practices.

**Results:**

Of the 258 surveyed doctors, 74 (28.7%) practiced ACP. Multiple logistic regression identified that age 20–49 years and experience in treating (or visiting facilities to treat) patients with COVID-19 infection were factors related to doctors that were significantly and positively associated with ACP practice. Additionally, a significant decline in the patients’ cognitive functions and difficulties in preventing COVID-19 infection were positively associated with ACP practiced by doctors.

**Conclusions:**

This study suggested that ACP should be practiced for older patients with dementia before COVID-19 infection, which would worsen their dementia symptoms. Moreover, ACP should be emphasized for older patients admitted to facilities. Our results could help devise effective measures to facilitate ACP practices.

**Supplementary Information:**

The online version contains supplementary material available at 10.1007/s40520-025-03004-9.

## Introduction

Since the end of 2019, the novel coronavirus disease (COVID-19) has become a worldwide threat, causing unfavorable outcomes. The mortality rate was high for vulnerable older adults, with outbreaks occurring in care facilities and hospitals where they gathered or stayed, reporting numerous victims [1–3]. Moreover, quick deterioration in physical conditions after contracting COVID-19 infection caused the death of many older adults, without their wishes or intentions being fulfilled or seeing their families owing to COVID-19-related restrictions [4].

Advance care planning (ACP) is a process of providing care and support to adults of any age and at any stage of health by understanding and sharing their personal values, life goals, and preferences regarding future medical care [5]. The goal of ACP is to ensure that people receive medical care aligned with their values, goals, and preferences during serious and chronic illnesses [5]. ACP should be done before providing potential major acute lifesaving treatments, such as intensive care unit (ICU) admission or resuscitation [6].

Barriers to the adoption of ACP practices were observed during the COVID-19 outbreak. During this time, overburdened healthcare systems faced challenges as shortages of ICU beds and overwhelming loads left healthcare professionals with little time to engage in ACP discussions [7]. Additionally, physical distancing measures, including visitor restrictions in hospitals introduced to limit the spread of COVID-19, made it difficult for healthcare providers, patients, and their families to have in-person conversations [8]. These interactions are crucial for fostering trust and mutual understanding in sensitive discussions such as ACP [9]. These barriers limited the capacity for patients and their families to make informed decisions about their care, particularly in emergency or critical care settings. Moreover, older adults infected with COVID-19 tended to develop acute pneumonia and thrombosis, causing sudden deterioration of health; consequently, ACP could not be practiced [4, 10, 11]. In August 2020, to ensure that older adults receive medical care that is consistent with their values, goals, and preferences, the Japan Geriatrics Society (JGS) emphasized the importance of practicing ACP at earlier stagesbefore contracting the infection [7]. ACP is practiced by elderly care physicians or other physicians under the supervision of elderly care physicians. However, no study has examined the factors related to doctors who practice ACP [12].

Therefore, we investigated the ACP practice rate in geriatricians during the COVID-19 outbreak using a questionnaire survey. The factors related to ACP practices that would be useful in such situations were obtained from this study.

## Methods

### Study design and ethical considerations

This study employed a cross-sectional design. An anonymous online survey was conducted with geriatric specialists between October and December 2022, the period between the seventh and eighth waves of the COVID-19 outbreak in Japan. The survey was administered by Hiroshima University in collaboration with the COVID-19 response team of the JGS to assess the impact of COVID-19 experienced by JGS members. We also received permission from the JGS to conduct the anonymous online survey using the JGS mailing list. Because the method of this study was an anonymous questionnaire survey, approval by an ethics committee was not necessary according to the ethical guidelines of the Ministry of Health, Labour and Welfare (MHLW) [13]. The study was conducted in accordance with the principles of the Declaration of Helsinki.

### Participants

The participants received a link to the online questionnaire developed using Google Forms (Google Plex, Mountain View, CA, USA). Before participating in the survey, they were informed about the purpose, content, and privacy policy of the study: the survey was about the changes in geriatric medical care due to the COVID-19 pandemic from 2020 to the present day, targeting only JGS members, and did not collect personal information because it was an anonymous survey. They were informed that they would consent to participate in the study by anonymously answering the survey questionnaire, which was completed online.

### Measurements

The participants were asked about their demographic characteristics such as sex, age, specialization, institution type (general or private hospitals or clinics, long-term care facility, or others), prefecture (47 prefectures were divided into quartiles according to the cumulative number of COVID-19 cases at the time of the survey), and years of working as a doctor. Information about whether ACP was practiced or not was collected. The ACP definition was not explained. JGS members would be aware of the ACP definition because the JGS notified its members to encourage ACP practices during the COVID-19 pandemic [10]. Information regarding medical treatments of patients infected with COVID-19, difficulties encountered when caring for older patients compared to that of younger patients with the infection, and treatment of patients with sequelae of the infection during the pandemic in their clinical practice were collected through a questionnaire with multiple or single answer options. Details of the questionnaire are presented in Supplementary Table 1.

### Statistical analysis

The participants’ characteristics were summarized according to the presence or absence of ACP practices. To compare the categorical variables between those with and without ACP practice, a χ2-test or Fisher’s exact test was performed.

To determine the significant factors associated with ACP practice, models 1 and 2 were obtained using multiple logistic regression (forward stepwise selection). In connection with relationships between the characteristics of doctors and ACP practice, independent variables—sex (male or female), age group (< 50 or ≥ 50 years), specialization, type of institution they were affiliated with, prefecture, and years worked—were added to the regression models. In addition, to determine whether the type of patients a doctor treats and difficulties in treating patients are related to ACP practice, the variables “medical treatment of patients with COVID-19 infection,” “difficulties encountered when caring for older patients with COVID-19 infection,” and “treatments of patients with sequelae of COVID-19 infection” were also included in the models.

In model 1, the experience as in-charge of treating patients with COVID-19 infection was added using the variable, “treating patients with COVID-19 infection,” which was defined as care provided in at least one of the following settings: “visiting facilities,” “visiting patients’ homes,” and “COVID-19 wards.” To delineate the most strongly related variable to ACP practice in model 2, the three variables were entered instead of “treating patients with COVID-19 infection.”

All analyses were performed using the IBM SPSS Statistics 28. The significance level was *p* < 0.05.

## Results

Among approximately 6,400 JGS members, 4,197 were on the mailing list at the time of the survey and 414 responded to the survey (response rate, 9.9%). Among them, 264 responded to the questionnaire regarding ACP practices. Six participants were excluded because they were not involved in treating patients with COVID-19 infection. Finally, 258 participants were included in the analysis.

The participants’ characteristics are presented in Table 1. Of the 258 participants, 74 (28.7%) engaged in ACP. Overall, the proportion of men was high (73.3%). There was a significant difference only in the age group; the proportion of those aged 20–49 years was higher in the ACP-practice group (44.6%) than in the no-ACP-practice group (22.3%; *p* < 0.001).

**Table 1:** Characteristics of participants

		All (*n* = 258)	ACP-practice (*n* = 74)	No-ACP-practice (*n* = 184)	*p*
		*N* (%)	*N* (%)	*N* (%)	
Sex					0.318
	Male	189 (73.3)	51 (68.9)	138 (75.0)	
	Female	69 (26.7)	23 (31.1)	46 (25.0)	
Age group					< 0.001
	20 − 49 years	74 (28.7)	33 (44.6)	41 (22.3)	
	50 ≥ years	184 (71.3)	41 (55.4)	143 (77.7)	
Specialization					0.146
	Geriatric or geriatric medicine	74 (28.7)	24 (32.4)	50 (27.2)	
	General internal medicine	113 (43.8)	36 (48.6)	77 (41.8)	
	General surgery or others	71 (27.5)	14 (18.9)	57 (31.0)	
Institution type					0.800
	General or Private hospitals	160 (62.0)	45 (60.8)	115 (62.5)	
	Clinics, Long term care facilities, or others	98 (38.0)	29 (39.2)	69 (37.5)	
Prefecture^†^					0.408
	First quartile	46 (17.8)	10 (13.5)	36 (19.6)	
	Second quartile	51 (19.8)	12 (16.2)	39 (21.2)	
	Third quartile	43 (16.7)	13 (17.6)	30 (16.3)	
	Fourth quartile	118 (45.7)	39 (52.7)	79 (42.9)	
Years worked					0.266
	1 − 10 years	27 (10.5)	7 (9.5)	20 (10.9)	
	11 − 30 years	126 (48.8)	42 (56.8)	84 (45.7)	
	31 ≥ years	105 (40.7)	25 (33.9)	80 (43.5)	

^†^Forty-seven prefectures were divided into quartiles according to the cumulative number of COVID-19 cases at the time of the survey

*P*-values were calculated using a chi-square test or Fisher’s exact test

ACP, advance care planning

Table 2 lists the differences in all the variables between the two groups. Regarding medical treatments of patients with COVID-19 infection, not only “treating patients with COVID-19 infection,” but also “visiting facilities,” “visiting patients’ homes,” and “COVID-19 infected wards” were significantly higher in the ACP-practice group than in the no-ACP-practice group (*p* < 0.05). In difficulties encountered when caring for older patients with COVID-19 infection, the ACP-practice group had high rates of “a high incidence of delirium” (40.5%), “significant decline in cognitive functions” (60.8%), and “difficulty in preventing COVID-19 infection (64.9%), such as isolation due to a lack of cooperation of patients,” compared to the no-ACP-practice group (*p* < 0.01). The ACP-practice group treated more patients with sequelae of COVID-19 infection than the no-ACP-practice group (*p* = 0.004).

**Table 2:** Differences in each variable between ACP-practice and no-ACP-practice groups

		All (*n* = 258)	ACP-practice (*n* = 74)	No-ACP-practice (*n* = 184)	*p*
		*N* (%)	*N* (%)	*N* (%)	
Medical treatments of patients with COVID-19					
	Diagnosed COVID-19 based on fever in outpatient clinics	154 (59.7)	44 (59.5)	110 (59.8)	0.962
	Treating patients with COVID-19 infection	133 (51.5)	51 (68.9)	82 (44.6)	< 0.001
	Visiting facilities	79 (30.6)	31 (41.9)	48 (26.1)	0.013
	Visiting patients’ homes	34 (13.2)	16 (21.6)	18 (9.8)	0.011
	COVID-19 wards	59 (22.9)	25 (33.8)	34 (18.5)	0.008
	Treating patients with COVID-19 admitted at COVID-19 infected wards as a consultant	19 (7.4)	6 (8.1)	13 (7.1)	0.772
Difficulties encountered when caring for older patients with COVID-19 infection					
	High incidence of delirium	70 (27.1)	30 (40.5)	40 (21.7)	0.002
	Significant decline in physical function	200 (77.5)	63 (85.1)	137 (74.5)	0.063
	Significant decline in cognitive functions	122 (47.3)	45 (60.8)	77 (41.8)	0.006
	Difficulties in preventing COVID-19 infection, such as implementing isolation due to a lack of cooperation from patients	129 (50.0)	48 (64.9)	81 (44.0)	0.002
	Difficulties in coordinating discharge of patients	84 (32.6)	28 (37.8)	56 (30.4)	0.251
	Difficulties in securing intermediate facilities for rehabilitation when hospitalized patients recovered	54 (20.9)	20 (27.0)	34 (18.5)	0.127
Treatments of patients with sequelae of COVID-19 infection					0.004
	Yes	130 (50.4)	48 (64.9)	82 (44.6)	
	No	96 (37.2)	16 (21.6)	80 (43.5)	
	Do not know	32 (12.4)	10 (13.5)	22 (12.0)	

*P*-values were calculated using a chi-square test or Fisher’s exact test

ACP, advance care planning

**Fig. 1:**
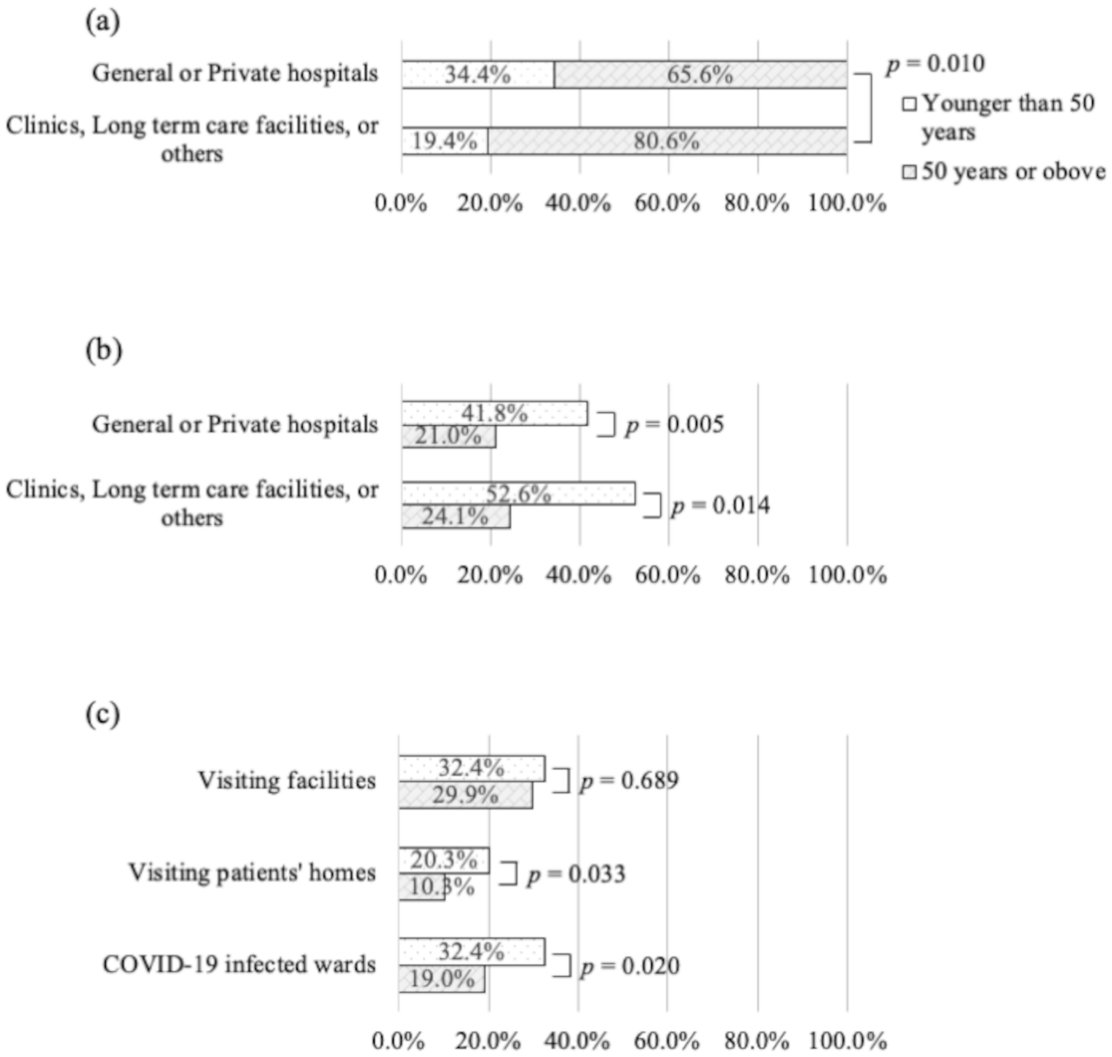
Proportions of the age range of participants. (**a**) Institution types, (**b**) ACP practice by institution type, and (**c**) Treating patients with COVID-19 infection. The *p*-values were calculated using a chi-square test. Multiplicity was adjusted using the Bonferroni method. ACP, advance care planning

More participants aged 20–49 years than those aged 50 years and above attended general or private hospitals and practiced ACP, regardless of the institution type. In contrast, in ACP practices at facilities, there was no significant difference by age group, whereas higher proportions of participants aged 20‒49 years mentioned “visiting patients’ homes” and “COVID-19 infected wards” (Fig. 1).

The results of the multiple logistic regression analyses are presented in Table 3. In model 1, age “20–49 years,” “treating patients with COVID-19 infection,” “significant decline in cognitive functions,” and “difficulty in preventing COVID-19 infection, such as isolation due to the lack of cooperation of patients,” were significant independent variables. In model 2, instead of “treating patients with COVID-19 infection,” “visiting facilities” was significant.

**Table 3:** Results of multiple logistic regression analyses (forward selection) using ACP practice as a dependent variable

Independent variables	Model 1			*P*	Model 2			*p*
	OR	95% CI			OR	95% CI		
		Lower limit	Upper limit			Lower limit	Upper limit	
Age group (Reference: 50 ≥ years)								
20 − 49 years	2.852	1.539	5.283	< 0.001	2.886	1.566	5.319	< 0.001
Treating patients with COVID-19 infection	2.356	1.289	4.308	0.005	-	-	-	-
Visiting facilities	-	-	-	-	1.929	1.056	3.524	0.033
Significant decline in cognitive functions	2.195	1.216	3.963	0.009	2.364	1.314	4.252	0.004
Difficulties in preventing COVID-19 infection, such as implementing isolation due to a lack of cooperation from patients	1.868	1.031	3.386	0.039	1.980	1.099	3.567	0.023

Model 1 included “treating patients with COVID-19 infection” and Model 2 included visiting facilities, visiting patients’ homes, and COVID-19 infected wards instead of “treating patients with COVID-19” as independent variables

ACP, advance care planning; CI, confidence interval; OR, odds ratio

## Discussion

We investigated the rate of ACP practice during the COVID-19 outbreak by surveying doctors who treated older patients with COVID-19 infection. Doctors aged 20‒49 years comprised a higher proportion of the ACP-practice group than those aged 50 years or more. The significant factors related to engagement in ACP practice were age “20–49 years,” “treating patients with COVID-19 infection (or visiting facilities),” “significant decline in cognitive functions,” and “difficulties in preventing COVID-19, such as isolation due to the lack of cooperation of patients.”

To the best of our knowledge, only two previous studies have investigated the rate of ACP practice in Japan during the COVID-19 pandemic. One study reported a rate of only 7% among hospitalized patients with COVID-19 infection aged 60–99 years; however, the results were limited to inpatients in an academic medical center and a rural acute care hospital and did not examine the factors related to doctors [14]. Similarly, another study using a web-based survey of residents aged 20–84 years reported that 9.4% of the respondents had discussed ACP [15]. In contrast, our study is the first to conduct a survey among doctors regarding their engagement in ACP practices during the COVID-19 period. In our study, the rate of ACP practice during the COVID-19 pandemic was 28.6%, similar to that (27.3%) reported by the MHLW in 2018 [16]. Unlike the MHLW report, which covered doctors in all fields but did not include doctors in clinics or care nursing homes, this study’s sample included doctors working in care nursing homes. Therefore, it was difficult to compare the ACP practice rate between the results of this study and that of the MHLW report; however, our study demonstrated that doctors have made efforts to encourage patients to engage in ACP even during the COVID-19 pandemic.

ACP was strongly associated with the long-term relationship between primary care doctors and patients with serious illnesses through continuous care over time [17, 18]. Additionally, doctors who worked longer hours reported routinely engaging in ACP practices after adjusting the effects of the practice locations (rural, small town, suburb, and urban) [19]. In 2020, the MHLW in Japan reported that young doctors tended to work for more hours than older doctors, with weekly work hours ranging from 49 to 61 [20]. The trend also shows that the younger the doctor, the longer their work hours [20]. In Japan, young doctors tend to work at acute care hospitals and engage in clinical practice after completing their training. However, as they age, they are more likely to take on administrative roles [21]. In line with this context, this study demonstrated that young doctors practiced ACP more than older doctors in general or private hospitals, clinics, and long-term care facilities, indicating that ACP is practiced regardless of the work location. Moreover, a higher proportion of younger doctors compared to older doctors practiced ACP by visiting patients’ homes or COVID-19 infected wards.

Our study deduced that doctors who treated patients with COVID-19 infection engaged in ACP practices, especially by visiting facilities. This may have been affected by a newly-revised guideline by the MHLW [22] stating that ACP at the end of life needs to be promoted not only in hospitals but also in home medical care and long-term care facilities. The frequency of doctor visits to facilities varies across countries. A study of nursing home care in 30 countries found that in 37% of the countries, doctors regularly visited residents, while in another 37%, there were no doctor visits at all [23]. These findings highlight disparities in the availability of ACP practices in long-term care settings globally. In hospital settings, international data reveal substantial challenges in practicing ACP. For example, a study conducted in Taiwan indicated that the challenges of practicing ACP in hospitals were exacerbated by the overwhelming number of COVID-19 patients, placing additional strain on medical staff [24]. Similarly, a review of studies from the USA, UK, the Netherlands, Japan, and Taiwan identified systemic barriers, including insufficient time for discussions about ACP with other sectors such as palliative and long-term care, as well as restrictions on hospital visits by relatives [25]. In contrast, community-based ACP has been prioritized for older adults admitted to facilities [25]. These settings allow patients more time to discuss their values and care preferences with family members and healthcare professionals before contracting COVID-19 [25, 26]. This suggests that community-based approaches to ACP may be more effective in fostering meaningful and proactive conversations, especially in less time-constrained environments. Nevertheless, further studies are necessary to determine the extent to which differences in where ACP is practiced are affected by COVID-19.

Most ACP practices were initiated after a diagnosis of dementia [27]. Caregivers of older adults with dementia reported difficulties in making older adults follow social restrictions to prevent infection with COVID-19 during the COVID-19 pandemic [28]. In addition, dementia symptoms worsened with the increase in the social restriction period [29]. A significant decline in the cognitive functions of patients and difficulty in preventing COVID-19, due to the lack of cooperation from patients in following isolation rules, were related to ACP practices in this study. It is presumed that doctors who recognized these symptoms practiced ACP understanding that ACP would not be possible if dementia worsened. These emphasized the importance of ACP practices for older adults with dementia before infection with COVID-19 because they were at a greater risk of developing delirium and worsened behavioral and psychological symptoms of dementia [29, 30].

This study has important implications in that it reveals the rate of ACP by doctors during the COVID-19 pandemic as the JGS emphasized the importance of implementing ACP in 2020, when COVID-19 spread globally [10]. The ACP rate was maintained by the workload of the young doctors. However, by April 2024, a new system for reforming how doctors work in Japan became effective, reducing their working hours [31]. Therefore, it is anticipated that the rate of ACP will not be maintained because of reduced working hours. This implies that other measures and support should be devised to maintain the rate of ACP regardless of doctors’ working hours and promote ACP to doctors of difference ages.

This study had several limitations. First, the longitudinal change in the ACP rate from the beginning of the COVID-19 outbreak to the present could not be determined because our study had a cross-sectional design. Second, the study’s response rate (9.9%) is relatively low. Among the JGS members, there were fewer than 1,500 board-certified geriatricians and many non-physician professionals [32]. This survey was primarily conducted for physicians, which may have contributed to the low response rate. Nevertheless, the findings may not fully represent all geriatricians in Japan or be generalizable to the entire population. Therefore, the results should be interpreted with caution, particularly regarding the doctors who did not respond to the survey. Third, this study did not investigate ACP practices in medical situations or clinical conditions. A gap in ACP practice may exist between an earlier stage and the end-of-life stage. In addition, ACP in cases of chronic diseases among older adults has mainly been implemented; however, it is difficult to implement ACP for acute-phase diseases such as pneumonia. Therefore, it was unclear whether ACP was practiced because of factors related to COVID-19 or factors other than COVID-19. Fourth, specific components of ACP, such as doctors’ practices related to end-of-life preferences or resuscitation decisions, were not analyzed. A retrospective study during the COVID-19 pandemic found that documented ACP was linked to “Do-Not-Resuscitate” orders, ICU admissions, mechanical ventilation, discharge to facilities or hospice, and death [33]. However, this survey was conducted in various settings, including nursing care facilities and hospitals. Therefore, the content of the ACP implemented is likely to vary significantly depending on the location. Fifth, challenges associated with institutional support for ACP during the pandemic were not explored. These challenges include time constraints, high patient workloads limiting clinicians’ ability to conduct meaningful ACP discussions, insufficient training for healthcare providers, and the ethical complexities of ACP documentation [34]. Infection control measures that restricted in-person conversations, necessitating a shift to telehealth solutions, were also not accounted for in this study [34]. Moreover, the roles of other healthcare professionals, such as nurses and social workers—essential for fostering patient and family inclusion, understanding prognosis, and preventing overtreatment—were not examined here [35]. In addition, the role of family members in ACP decision-making remains underexplored. Family members provide essential psychological, emotional, and practical support and play a critical role in end-of-life care, particularly in ensuring patient preferences are honored [36]. During the pandemic, family caregivers encountered significant barriers, including care plan disruptions, caregiver burden, isolation that limited ACP engagement, and a lack of knowledge about ACP, all of which may have hindered their ability to actively participate in ACP discussions [37]. Sixth, the investigation did not address the precise timing of ACP assessments. The initiation of ACP during the pandemic was often driven by acute clinical deterioration or the onset of critical illness [38]. Institutional barriers mentioned above and individual-level challenges, such as lack of consumer knowledge about ACP, perceived irrelevance of ACP, lack of trust, and cultural reluctance to discuss death, likely influenced the timing of discussions [38]. Rapid deterioration often left insufficient time for meaningful discussions [38]. Interpersonal-level barriers, such as role ambiguity between doctor and patient expectations, social distancing prohibiting face-to-face conversations, and family shock and grief further complicated ACP timing [38]. Finally, the diagnosis of dementia and fluctuations in cognitive function was dependent on the judgment of each respondent, which may have influenced the results. Furthermore, since the feasibility of ACP by individuals is affected by cognitive function, this may also have impacted the results.

## Conclusions

This study revealed that the factors related to engagement in ACP were the age of the doctors who treated (or visited facilities to treat) patients with COVID-19 infection and dementia, worsening symptoms of dementia, and difficulty in implementing isolation owing to the lack of cooperation of patients. Based on the results of this study, effective measures for coping with these factors can be devised.

## Electronic supplementary material

Below is the link to the electronic supplementary material.

Supplementary Material 1

## Data Availability

No datasets were generated or analysed during the current study.
