# Supplementary material for: Rate of advance care planning practice during the COVID-19 outbreak in Japan: a cross-sectional survey study
Source: Aging Clin Exp Res. 2025 Apr 7;37(1):116. doi: 10.1007/s40520-025-03004-9 (PMC11976364; doi:10.1007/s40520-025-03004-9)
Supplement: Supplementary file 1 — Supplementary Material 1 [file 40520_2025_3004_MOESM1_ESM.docx]

Supplement Table 1

| Question 1. What is your gender?  Select one that applies.  ○ Male  ○ Female  ○ Other  Question 2. What is your age?  Select one that applies.  ○ 20–29 years old  ○ 30–39 years old  ○ 40–49 years old  ○ 50–59 years old  ○ 60–69 years old  ○ 70–79 years old  ○ 80 years old or older  Question 3. What is your specialty?  Select one that applies.  ○ Geriatrics, Geriatric Internal Medicine  ○ General Internal Medicine  ○ General Surgery  ○ Other  Question 4. In what kind of facility do you work? (For those who work at multiple facilities, please indicate the kind of facility where you primarily work.)  Select one that applies.  ○ General hospital  ○ Private hospital  ○ Clinic  ○ Long-term care facility  ○ Other  Question 5. In what prefecture is your facility located?  Select one that applies.  ○ Hokkaido  ○ Aomori  ○ Iwate  ○ Miyagi  ○ Akita  ○ Yamagata  ○ Fukushima  ○ Ibaraki  ○ Tochigi  ○ Gunma  ○ Saitama  ○ Chiba  ○ Tokyo  ○ Kanagawa  ○ Niigata  ○ Toyama  ○ Ishikawa  ○ Fukui  ○ Yamanashi  ○ Nagano  ○ Gifu  ○ Shizuoka  ○ Aichi  ○ Mie  ○ Shiga  ○ Kyoto  ○ Osaka  ○ Hyogo  ○ Nara  ○ Wakayama  ○ Tottori  ○ Shimane  ○ Okayama  ○ Hiroshima  ○ Yamaguchi  ○ Tokushima  ○ Kagawa  ○ Ehime  ○ Kochi  ○ Fukuoka  ○ Saga  ○ Nagasaki  ○ Kumamoto  ○ Oita  ○ Miyazaki  ○ Kagoshima  ○ Okinawa  Question 6. How many years of experience do you have as a medical worker?  Select one that applies.  ○ 1–2 years  ○ 3–5 years  ○ 6–10 years  ○ 11–20 years  ○ 21–30 years  ○ 31–40 years  ○ 41–50 years  ○ 51 years or more  Question 7. Have you ever provided medical treatment for COVID-19? Please select all that apply.   Diagnosed COVID-19 based on fever in outpatient clinics.   Visited facilities to treat patients with COVID-19.   Visited patients’ homes to treat patients with COVID-19.   In charge of patients with COVID-19 in COVID-19 wards.   As a consultant, treated patients with COVID-19 who were admitted to COVID-19 infected wards.   Did not diagnose COVID-19   Others  Question 8. We would like to ask a doctor who has experience treating COVID-19 patients. Have you encountered difficulties in treating older patients than younger patients with COVID-19? Please select all that apply.   There was a high incidence of delirium in association with COVID-19.   There was a significant decline in physical function in association with COVID-19.   There was a significant decline in cognitive function in association with COVID-19.   It was difficult preventing the spread of COVID-19 infection, such as implementing isolation due to a lack of cooperation from patients.   It was difficult to coordinate discharge when hospitalized patients recovered.   It was difficult to secure intermediate facilities for rehabilitation when hospitalized patients recovered.  Question 9. We would like to ask a doctor who has experience treating patients with COVID-19. Have you ever performed Advance Care Planning discussion during your medical treatment?  ○ Yes.  ○ No.  Question 10. It is known that sequelae often exist after recovery from COVID-19  (It is generally considered sequelae that the symptoms persist for at least four weeks after the onset of the symptoms). Have you ever treated a patient with such sequelae?  Please select one that applies.  ○ Yes.  ○ No.  ○ Do not know. |
| --- |
